# Knowledge and Practices of South African Optometrists Regarding Childhood Myopia Management

**DOI:** 10.3390/vision10040063

**Published:** 2026-09-01

**Authors:** Shivani Naipal, Nishanee Rampersad, Rekha Hansraj

**Affiliations:** Discipline of Optometry, School of Health Sciences, University of KwaZulu-Natal, Private Bag X54001, Durban 4000, KwaZulu-Natal, South Africa; rampersadn@ukzn.ac.za (N.R.); hansrajr@ukzn.ac.za (R.H.)

**Keywords:** myopia control, myopia management, myopia progression, clinical practices, clinical decision-making

## Abstract

Myopia is a growing public health problem expected to affect half the global population by 2050, making appropriate management of the condition necessary. Therefore, the purpose of this study was to investigate the knowledge and practices of South African optometrists regarding childhood myopia management. A quantitative, cross-sectional, descriptive design with a survey method was employed. An online questionnaire was distributed to practicing optometrists and included questions related to demographic information, knowledge of myopia and associated ocular pathologies, diagnostic work-up and management of childhood myopia. Data were summarised using descriptive statistics, and further analysed with univariate binary and ordinal logistic regression analysis where the independent variables were years of experience, certification and interest. A total of 124 completed questionnaires were received. The majority of participants used non-cycloplegic refraction and less than 20% performed dilated fundus examinations. Single vision distance spectacles were most commonly prescribed despite awareness of the efficacy of interventions to slow myopia progression. However, factors such as cost and the need for clinical guidelines limit the practice of evidence-based management. Participants were aware of some of the ocular pathologies associated with high myopia, indicating that this knowledge needs to be reinforced. Results from this study may be used to aid optometry policy development, regulatory council decisions and guide the content of professional development programmes to enhance knowledge.

## 1. Introduction

Myopia is a growing public health problem expected to affect half of the global population by 2050 [1]. Similarly, in Southern Africa, the prevalence of myopia is expected to increase exponentially from 8% in 2010 to 30% by 2050 [1]. It should be noted that these estimates were made before the COVID-19 pandemic and may be higher owing to lifestyle and behavioural changes because of the COVID-19 pandemic [2,3]. A recent systematic review and meta-analysis concluded that there was a greater prevalence estimate of myopia in developing countries compared to developed countries [2]. As uncorrected refractive error is a major cause of vision impairment, the exponential increase in myopia will have considerable public health and socio-economic implications. These include limited educational and employment opportunities, loss of productivity and reduced quality of life [4].

Research shows that currently there is an earlier onset of myopia compared with previous generations [5]. This implies that there is a longer duration of living with myopia, greater risk of progression and heightened probability of vision impairment owing to sight-threatening conditions associated with high myopia [4]. With increasing concern about the rising prevalence of myopia and its associated conditions, there has been a shift from correcting myopia to managing myopia. Correcting myopia is characterised by the use of single-vision lenses or refractive surgery to correct the refractive error and provide clear vision [6]. This is in contrast to managing myopia, which involves a more holistic approach aimed at slowing the progression of myopia in addition to correcting the refractive error [6]. In April 2021, the World Council of Optometry (WCO) passed a resolution making myopia management the standard of care in optometric practice [7].

While studies have investigated knowledge and practices of eye-care practitioners regarding management of childhood myopia [8,9,10,11,12,13,14,15], there is limited information available within a South African context. A previous global study that investigated trends in myopia management in 2019 received a poor response from Africa and was therefore excluded from further analysis [8]. Despite an updated review in 2022 including more responses from Africa, the number was still inadequate to do a country comparison within Africa [15]. Another study investigating myopia management in Africa [11] also had a poor response from South Africa further underscoring the lack of information in a South African context. The higher prevalence of childhood myopia in East Asian countries such as China compared to in African countries such as Ghana and South Africa may explain the lower response rate from practitioners in African countries. However, Africa is undergoing rapid urbanisation, and research supports the link between increasing prevalence of myopia and increasing urbanisation [16]. In addition, a study investigating myopia prevalence in Africa found significantly higher prevalence rates in Ghana and South Africa than other African countries [17].

Optometrists in South Africa are regulated by the Health Professions Council of South Africa (HPCSA) and may be registered as being in independent practice, having diagnostic privileges (effective since 2001) or certified with ocular therapeutics (effective since 2015). To date, research on optometrists’ awareness and management practices regarding myopia in South Africa has been limited to a study conducted in KwaZulu-Natal [18]. However, no national study has comprehensively investigated the examination and management practices related to childhood myopia among South African optometrists. Therefore, it remains unknown whether the resolution passed by the WCO is being implemented in South Africa from the perspective of practicing optometrists. It is recognised that clinical care in developing countries such as South Africa is different from developed countries. For example, there is limited advanced equipment in primary-care settings in developing countries. Furthermore, there are limitations regarding management options available to South African optometrists specifically related to pharmacological options. The absence of baseline information affects optometry policy development and regulatory council decisions regarding resource allocations, standards of practice and evidence-based guidelines tailored for myopia management in the South African context. Therefore, the purpose of this study was to investigate the knowledge and practices of childhood myopia management among South African optometrists.

## 2. Materials and Methods

### 2.1. Research Setting

The study was conducted in South Africa and included registered optometrists who were actively practicing within the country at the time of data collection, irrespective of province, years of experience or practice setting (private, public, academic or corporate).

### 2.2. Study Design

This study employed a quantitative, descriptive, cross-sectional design. An online survey was distributed to registered optometrists practising in South Africa between March 2024 and February 2025 to investigate their knowledge and clinical practices relating to the examination and management of childhood myopia.

### 2.3. Study Population and Sampling Strategy

Participants were recruited using convenience sampling. There were 4204 optometrists registered with the HPCSA at study inception and only those practicing in South Africa were invited to participate. A sample size of 277 optometrists was calculated in consultation with a statistician using 80% power, 6% margin of error and an additional 10% to account for the possibility of drop-outs (Z Dessie, 2023, pers. comm., 23 May).

### 2.4. Data Collection

The online questionnaire was designed using Google forms and created such that questions appeared sequentially. Subsequent questions appeared only when a response to the previous question was submitted and there was no option to return to the previous question. All questions were mandatory and there was no option to skip any question. The questionnaire was distributed via national optometry organisations (South African Optometric Association and Graduate Institute of Optometry), newsletters (Eyesite.co.za) and social media platforms (Facebook, Instagram, WhatsApp and LinkedIn). A reminder to complete the questionnaire was sent a week after the initial invitation. All participants read an electronic information sheet that detailed the nature of the study and provided electronic informed consent prior to accessing the questionnaire. To aid in the detection of duplicate responses, the questionnaire contained a question that asked if this survey had been completed previously. As the questionnaire was adapted from a previous study [9], tests to assess validity were not conducted. The questionnaire was reviewed by the authors to assess whether the questions were context-appropriate and within the scope of practice for South African optometrists, and thereafter piloted among five optometrists to ensure efficiency and suitability. The responses from the pilot study were not included in the final study sample.

#### Study Questionnaire

A questionnaire adapted with permission from a previous study [9] was used. The questionnaire consisted of 33 questions divided into three sections that probed: (1) demographic information, (2) knowledge of myopia and its associated ocular pathologies, and (3) diagnostic work-up and management of childhood myopia. The questionnaire also contained a clinical scenario regarding cessation of myopia control based on age and duration of myopia stability. The questionnaire consisted of close-ended questions that allowed participants to select only one option, multiple options or rate on a five-point Likert scale. There was only one open-ended question where participants provided additional comments regarding childhood myopia management. A child was defined as aged 17 years or younger in the questionnaire. The estimated completion time was approximately 10 to 15 min.

### 2.5. Data Analysis

Data from the Google form were exported using Microsoft Excel and checked to ensure that all participants met the study criteria. Thereafter the Statistical Package for Social Sciences (SPSS version 30) was used for analysis. Descriptive statistics including frequencies and percentages were used to summarise the responses for each question. Univariate regression analysis was performed to determine an association between variables, and the odds ratio (OR) and 95% confidence interval (CI) are presented. Binary logistic regression was used to analyse survey questions with dichotomous responses and ordinal logistic regression was used for the Likert-scale questions. The independent variables were years of experience, therapeutics certified, ocular diagnostics techniques certified and clinical and research interest in myopia management, in line with previous research [9]. Years of experience was stratified into early career (0 to 5 years in practice) and experienced (more than 5 years) participants. Only results with acceptable goodness-of-fit parameters are presented. A probability (*p*) value of 0.05 or less was considered statistically significant. Responses to the open-ended question were imported into the qualitative data analysis software Nvivo (version 15), where it was coded and categorised into themes according to the principles of reflexive thematic analysis.

### 2.6. Ethical Considerations

Ethical approval was obtained from the Humanities and Social Sciences Research and Ethics Committee (HSSREC/00005846/2023) of the University of KwaZulu-Natal. Participants provided written informed consent before completing the questionnaire. No identifying data were collected thereby ensuring participant anonymity and confidentiality.

## 3. Results

### 3.1. Demographics

In total, 129 responses were received with 124 completed questionnaires, representing a response rate of 3%. Table 1 shows the demographic and practice characteristics of the participants (*n* = 124). Almost two-thirds of participants were female and just over 40% were aged 20–29 years (Table 1). More than one-third of participants had up to five years of optometric practice experience (*n* = 44). The majority of participants worked in independent (private) practice (*n* = 73) followed by corporate practice (*n* = 33). Most participants worked in urban areas with KwaZulu-Natal (*n* = 48) and Gauteng (*n* = 38) being most common (Table 1). While almost 90% of participants were certified for ocular diagnostics, less than 20% were ocular therapeutics certified. Furthermore, just over 70% of participants had either a clinical or research interest in managing childhood myopia. Almost two-thirds of participants (*n* = 80), the majority of whom were aged between 20 and 39 years, received myopia management education during their undergraduate studies. Approximately 60% of participants indicated providing care to up to five patients with childhood myopia in a typical week.

### 3.2. Clinical Routine and Diagnosis of Childhood Myopia

Table 2 shows the clinical procedures that participants performed routinely on children with myopia at first presentation. Over 75% of participants noted family history of myopia, performed non-cycloplegic subjective refraction and measured intraocular pressure. Cycloplegic refraction was performed less frequently comprising of cycloplegic retinoscopy (36%), cycloplegic subjective refraction (26%) and cycloplegic autorefraction (23%). A dilated retinal fundus examination was done by less than a quarter of participants while only 7% measured axial length. Other clinical procedures that were performed included an assessment of the anterior chamber angles, corneal pachymetry, macular optical coherence tomography, keratometry, ophthalmoscopy, suppression, colour vision and referral for a cycloplegic refraction. The majority of participants (*n* = 87) indicated routinely referring patients to ophthalmologists for monitoring of ocular health. Similarly, the majority of participants (*n* = 79) indicated that they collaborate with ophthalmologists to manage childhood myopia. Non-cycloplegic retinoscopy was more likely to be performed by experienced clinicians (OR 2.22, 95% CI 1.01–4.90, *p* = 0.048). Participants with ocular diagnostics certification were more likely to measure intraocular pressure (OR 3.27, 95% CI 1.07–9.97, *p* = 0.037).

**Figure 1:**
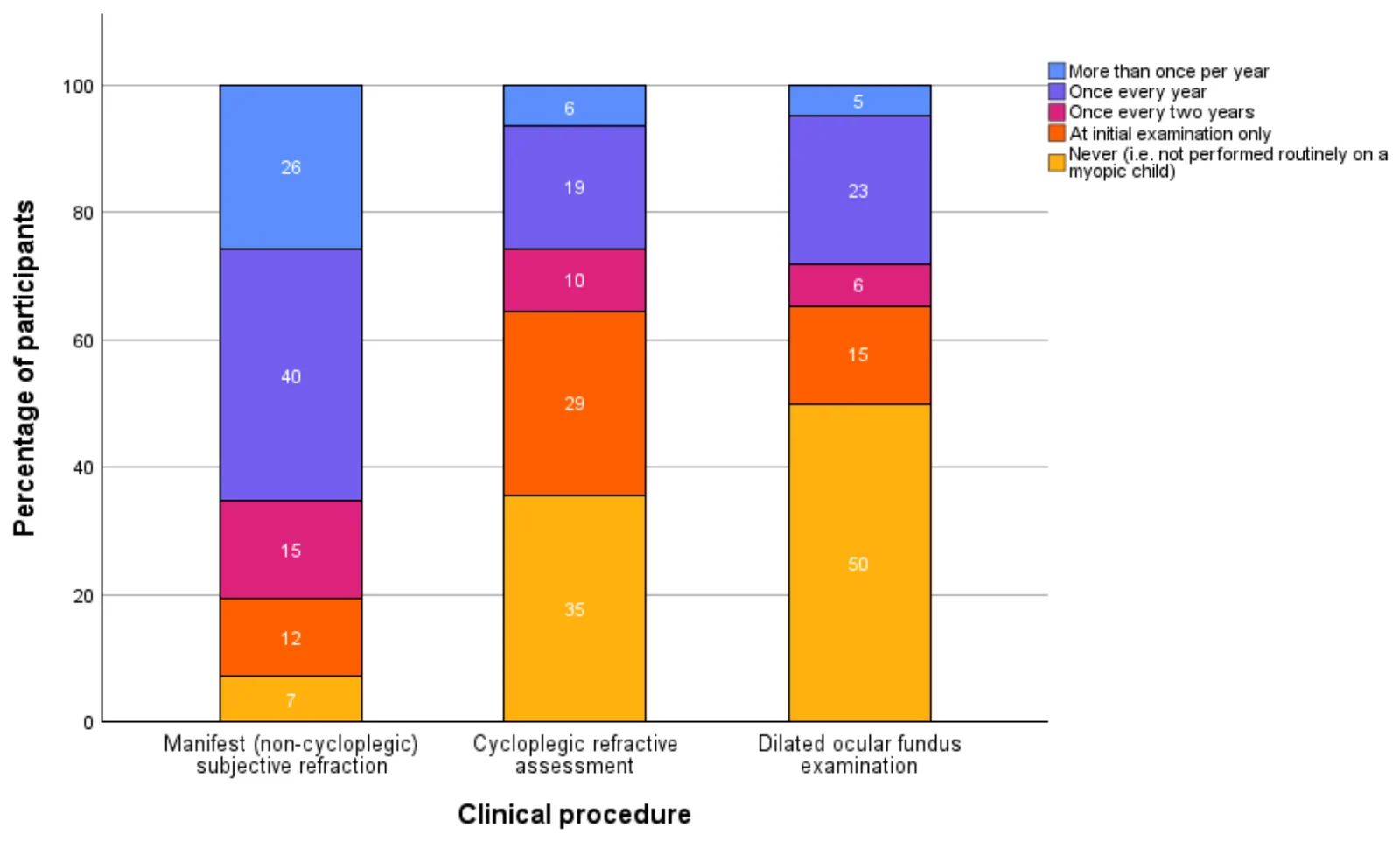
Percentage of responses for clinical procedures performed routinely on a child with myopia.

Cycloplegic refraction and dilated ocular fundus examination were never routinely performed on a child with myopia by almost one-third (35%) and half (50%) of participants, respectively (Figure 1). Participants were more inclined to perform manifest (non-cycloplegic) subjective refraction routinely, with almost a quarter (26%) of participants performing a manifest refraction more than once a year on a child with myopia. Furthermore, participants with a clinical (OR 5.76, 95% CI 1.92–17.29, *p* = 0.002) or research (OR 1.99, 95% CI 1.00–3.96, *p* = 0.049) interest in managing childhood myopia were more likely to perform manifest (non-cycloplegic) subjective refraction.

### 3.3. Knowledge of Ocular Complications Associated with High Myopia

Participants’ responses regarding ocular conditions associated with high myopia are presented in Table 3. More than half the participants identified retinal breaks, rhegmatogenous retinal detachment and primary open-angle glaucoma as complications of high myopia while less than 50% indicated an association between cataract and high myopia. Only 13% of participants (*n* = 16) were able to correctly identify all ocular conditions associated with high myopia. Participants certified with ocular diagnostics were more likely to identify retinal breaks (OR 3.69, 95% CI 1.09–12.50, *p* = 0.036) and primary open-angle glaucoma (OR 6.38, 95% CI 1.70–23.95, *p* = 0.006) as being associated with high myopia.

### 3.4. Perceived Efficacy of Myopia Control Interventions

Table 4 shows participant responses regarding myopia control interventions considered as effective for slowing childhood myopia progression. The majority of participants (*n* = 112) considered advice to increase time spent outdoors to be effective in slowing childhood myopia progression. This was followed by low-dose (0.01–0.5%) atropine eye drops (*n* = 94) and visual hygiene such as taking regular breaks during prolonged near work (*n* = 91). Almost two-thirds of participants considered orthokeratology and peripheral defocus soft contact lenses as effective while almost 40% indicated full correction single vision distance spectacles as effective. Other myopia control interventions considered effective included accommodative support lenses, peripheral-defocus spectacle lenses, such as the defocus-incorporated multiple-segments (DIMSs) lenses, and corneal cross-linking (Table 4). Participants’ responses varied regarding the ranking order of the different interventions considered most effective for myopia control. The intervention that ranked highest was low-dose atropine eye drops (*n* = 25) followed by peripheral-defocus soft-contact lenses (*n* = 18) and orthokeratology (*n* = 17). Participants with ocular diagnostics were more likely to select low-dose atropine (OR 3.27, 95% CI 1.07–9.97, *p* = 0.037) and peripheral-defocus soft-contact lenses (OR 3.46, 95% CI 1.14–10.49, *p* = 0.029). Participants with a clinical interest in myopia management were more likely to select orthokeratology (OR 6.47, 95% CI 1.61–25.89, *p* = 0.008) and peripheral-defocus soft-contact lenses (OR 6.20, 95% CI 1.55–24.79, *p* = 0.010). Experienced participants were more likely to select progressive-addition spectacle lenses (OR 2.31, 95% CI 1.09–4.90, *p* = 0.029) and less likely to select full-correction single-vision distance spectacles (OR 0.42, 95% CI 0.20–0.89, *p* = 0.023).

### 3.5. Factors Influencing Management

**Figure 2:**
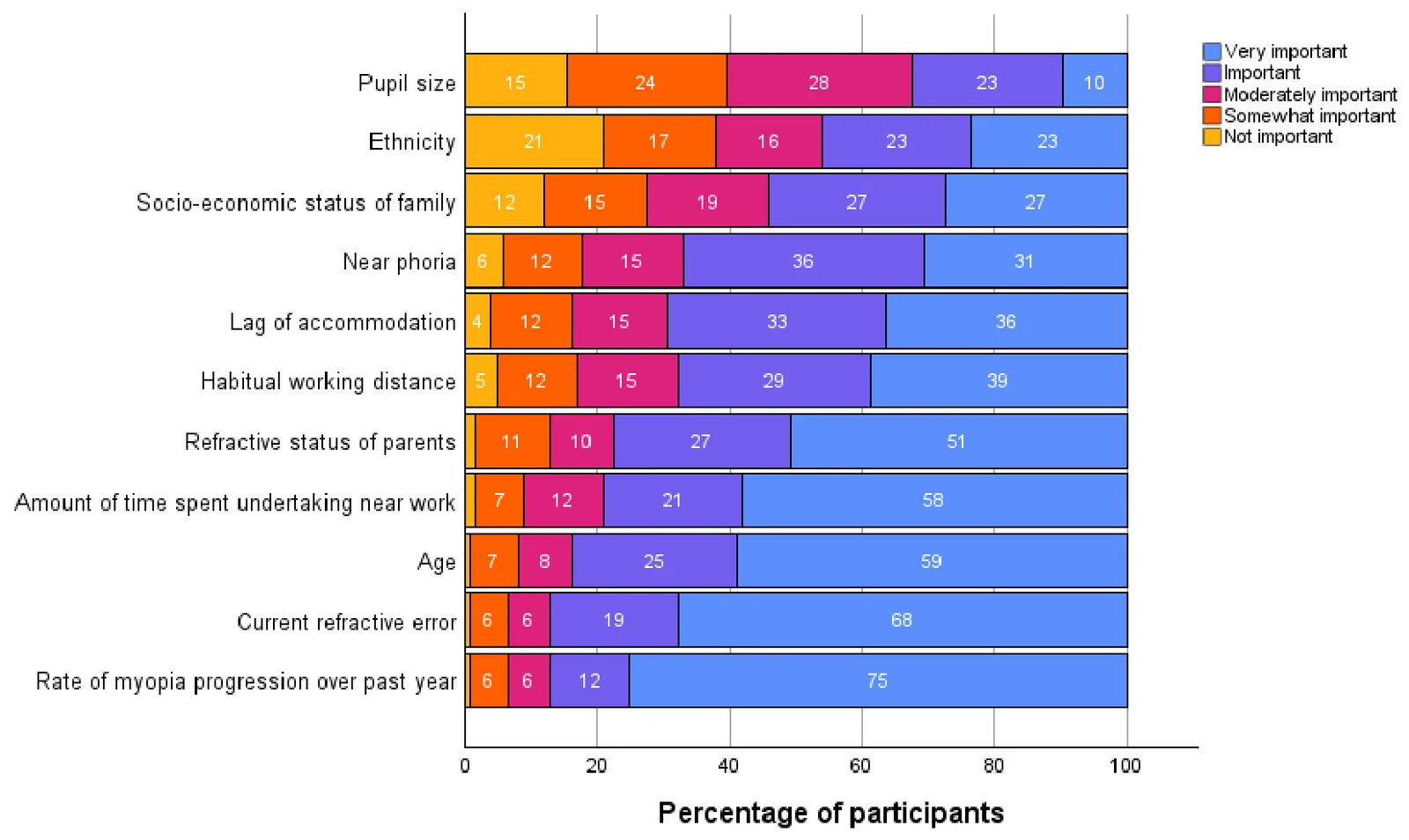
Percentage of responses rating the relative importance of various factors pertaining to the patient when deciding on the management approach for a child with myopia.

Figure 2 shows participants’ responses regarding the relative importance of several factors pertaining to the patient when deciding on the management approach for a child with myopia. The majority of participants (*n* = 93) considered the rate of myopia progression over the past year to be very important. This was followed by current refractive error (*n* = 84), age (*n* = 73) and amount of time spent undertaking near work (*n* = 72). Ethnicity (*n* = 26), pupil size (*n* = 19) and socio-economic status (*n* = 15) were considered to be least important. Participants with a clinical interest in managing childhood myopia were more likely to consider the refractive status of the patient’s parents (OR 4.68, 95% CI 1.53–14.28, *p* = 0.007) while this was not a consideration for experienced participants (OR 0.29, 95% CI 0.14–0.60, *p* = 0.001). Participants with a research interest in managing childhood myopia were more likely to consider the amount of time spent undertaking near work as important (OR 2.08, 95% CI 1.00–4.29, *p* = 0.049).

Responses related to the relative importance of factors that currently limit the management of childhood myopia are shown in Supplementary Figure S1. The need to purchase additional clinical equipment and minimal financial incentive were rated as very important limitations by more than 20% of participants. Experienced participants were less likely to consider the need to purchase additional clinical equipment (OR 0.33, 95% CI 0.17–0.64, *p* = 0.001) and minimum financial incentives (OR 0.36, 95% CI 0.18–0.71, *p* = 0.003) as important limitations. Concern over medico-legal aspects of prescribing interventions other than spectacles and lack of regulatory approval of treatments for slowing myopia progression were rated as unimportant by less than 20% of participants. Participants with a clinical interest in managing childhood myopia were less likely to consider medico-legal aspects (OR 0.30, 95% CI 0.10–0.93, *p* = 0.037) and time for professional development (OR 0.30, 95% CI 0.10–0.87, *p* = 0.026) as important. Lack of interest in managing myopia was less likely to be important for participants with a clinical interest (OR 0.16, 95% CI 0.05–0.48, *p* = 0.001) and research interest (OR 0.38, 95% CI 0.19–0.78, *p* = 0.008) in managing childhood myopia.

### 3.6. Management of Childhood Myopia

**Figure 3:**
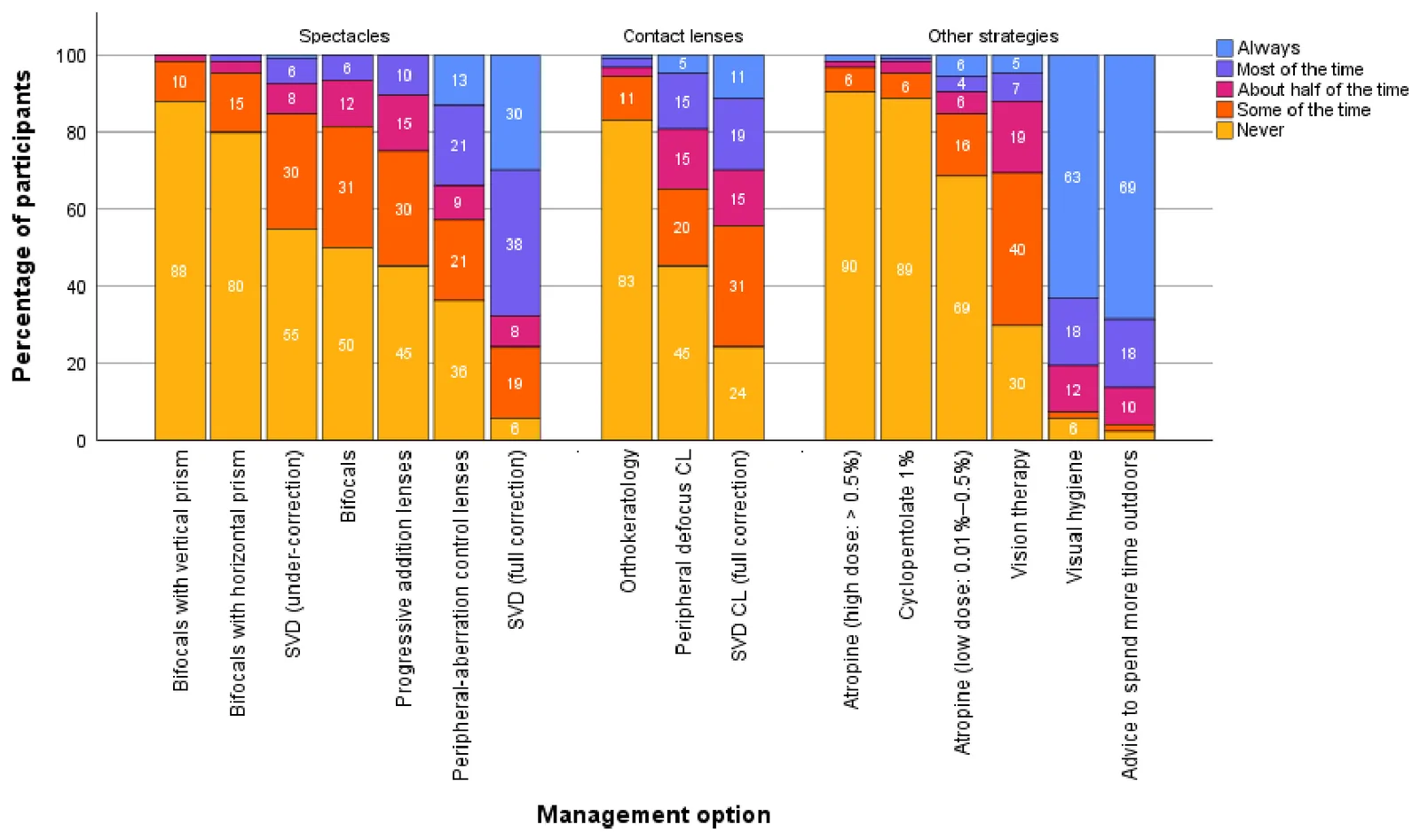
Percentage of responses for management options prescribed to children with myopia.

More than 50% of participants (*n* = 69) indicated that the minimum spherical equivalent myopic correction that they would prescribe to a child was −0.50 D, while almost a quarter of participants (*n* = 30) indicated prescribing a minimum spherical equivalent myopic correction of −0.75 D. Figure 3 shows participant responses for management options prescribed for children with myopia. Single-vision distance spectacles (full correction) were most frequently prescribed, with 33% and 40% of participants prescribing them always and most of the time, respectively. However, participants with a clinical interest in managing childhood myopia were more likely to prescribe peripheral aberration control spectacle lenses (OR 6.70, 95% CI 1.75–25.62, *p* = 0.005). For the contact lens options, single-vision distance (full correction) was the most frequently prescribed. Orthokeratology, peripheral-defocus contact lenses and low-dose atropine eye drops were not frequently prescribed (Figure 3). However, participants with therapeutics certification were more likely to prescribe low-dose atropine eye drops (OR 2.61, 95% CI 1.10–6.19, *p* = 0.030). Almost two-thirds of participants always advised patients to spend more time outdoors (*n* = 85) and to exercise visual hygiene (*n* = 78), with those participants with a research interest being more likely to advise patients to increase time spent outdoors (OR 3.45, 95% CI 1.57–7.56, *p* = 0.002).

Supplementary Table S1 shows the responses for the clinical scenario regarding the cessation of myopia control based on age and duration of myopia stability. Responses regarding the age varied among participants and almost 30% indicated that it would be appropriate to cease active treatment at 21 years, while 24% and 16% indicated 18 and 16 years, respectively. Almost one-fifth of participants stated that active treatment could be ceased at any age. The majority of participants (*n* = 57) indicated that they would cease active treatment if there was no progression of myopia for 24 months while almost 30% stated that myopia needed to be stable for 12 months before ceasing treatment. The most frequent response was to cease active treatment at 21 years if no progression of myopia for 24 months was noted (*n* = 22).

### 3.7. Sources of Information

**Figure 4:**
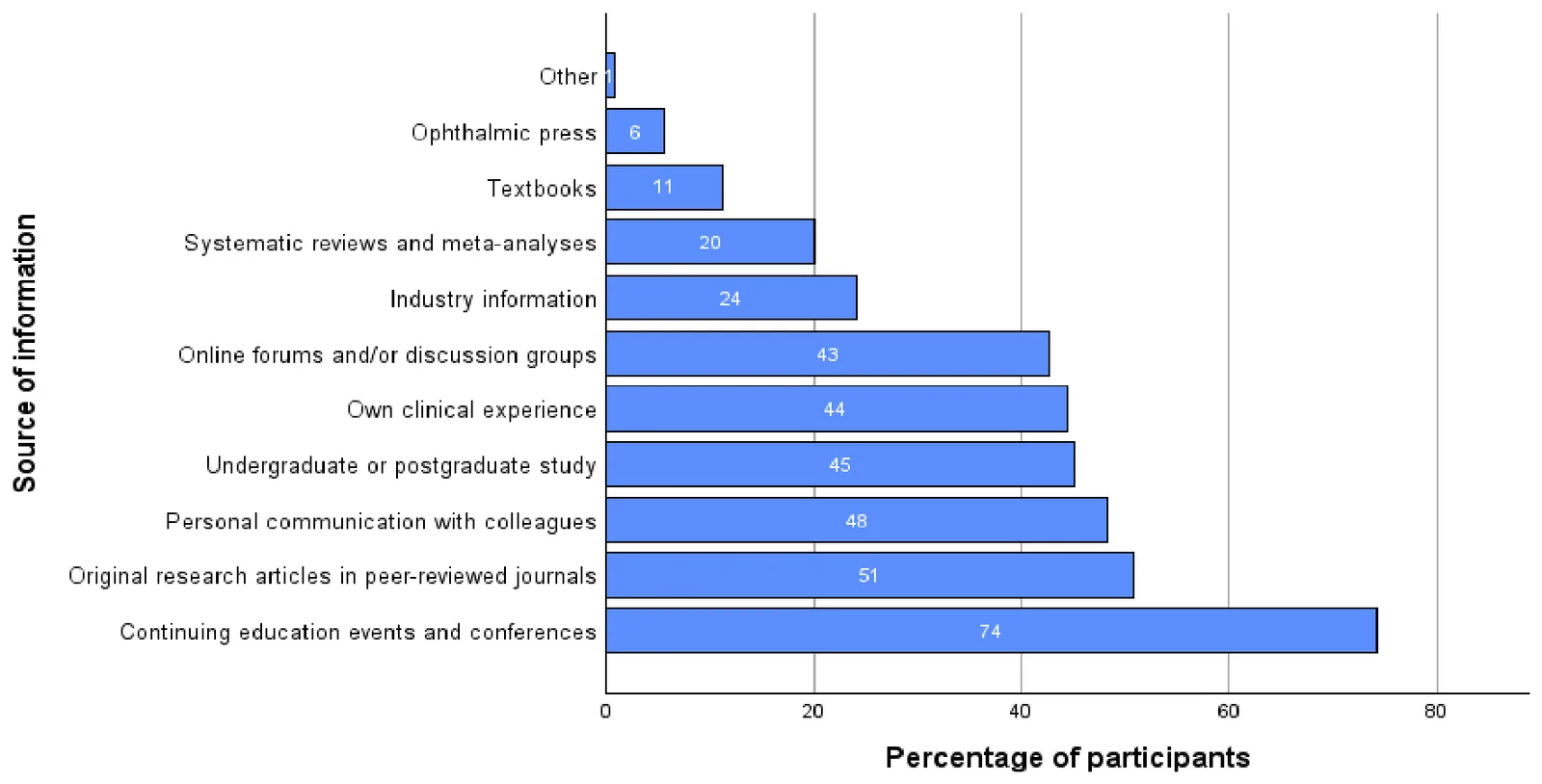
Percentage of responses regarding sources of information that influenced perception of effective myopia management options.

Figure 4 shows the sources of information that influenced participants’ perceptions of the most effective methods for myopia management. The majority of participants (*n* = 92) relied on continuing education events and conferences followed by original research articles in peer-reviewed journals (*n* = 63) and personal communication with colleagues (*n* = 60). More than 40% of participants used online forums and/or discussion groups, their own clinical experience as well as their undergraduate or postgraduate study. Another source, mentioned by one participant, was an online myopia-management course. Systematic reviews and meta-analyses were more likely to be selected by participants with a research interest in managing childhood myopia (OR 3.84, 95% CI 1.07–13.74, *p* = 0.039).

Supplementary Figure S2 shows participants’ ratings of the relative importance of various resources to guide their approach to managing childhood myopia. Overall, all resources listed in the figure were considered important by participants in guiding their approach to myopia management. All participants considered their own clinical experience to be important to varying degrees. Almost 60% of participants considered continuing education events and conferences and original research articles in peer-reviewed journals to be very important.

Supplementary Figure S3 shows participants’ ratings of the relative usefulness of various sources in supporting their clinical management of childhood myopia. The majority of participants (*n* = 85) considered clinical tools based on best-available research evidence to be very useful. This was followed by clinical guidelines for childhood myopia management that outlined best-practice clinical care (*n* = 79) and a free website to promote the dissemination of new research evidence (*n* = 77). Less than 10% of participants (*n* = 8) considered the use of online subscriptions to independently access scientific journal articles as not useful.

### 3.8. Engagement with Caregivers of Children with Myopia

**Figure 5:**
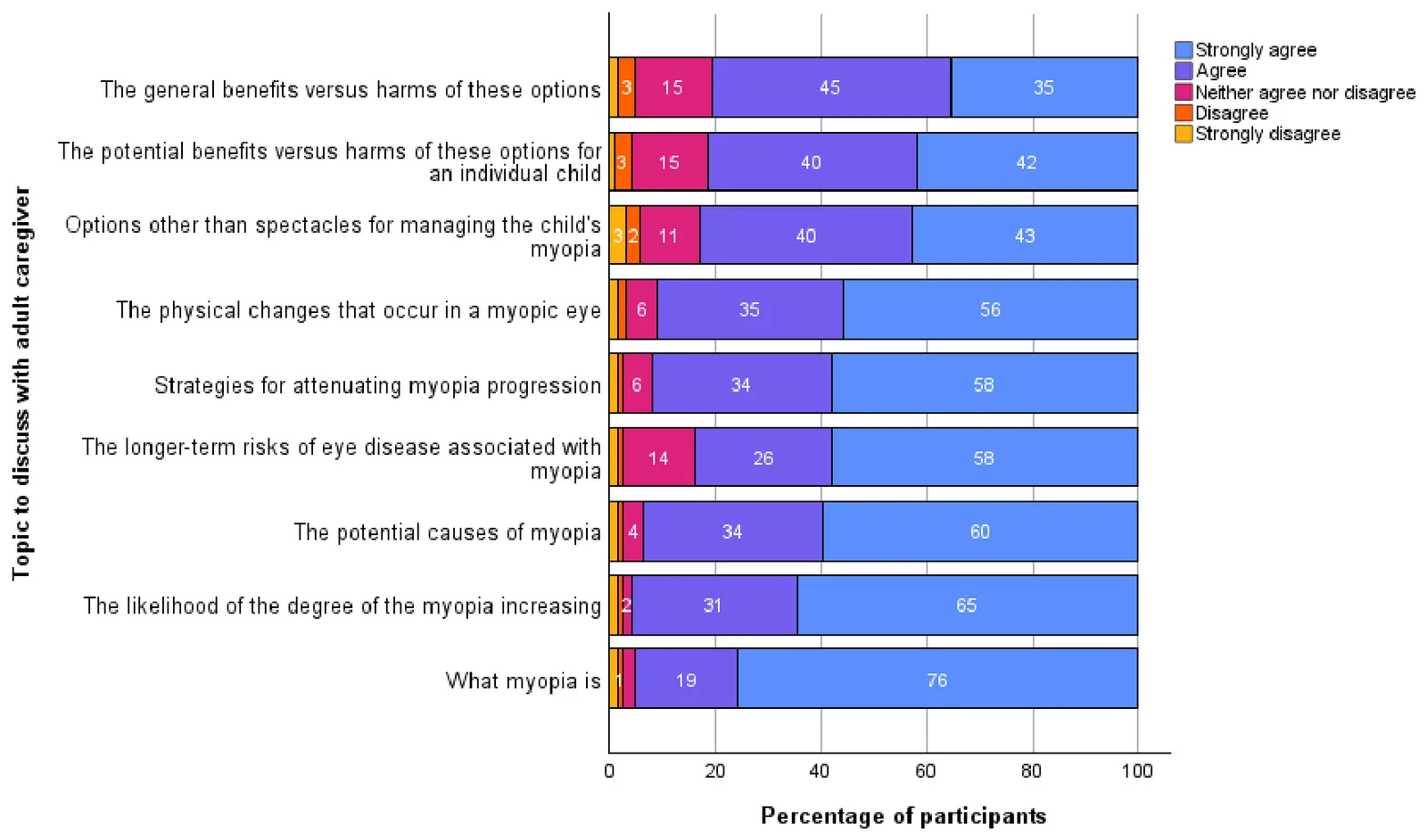
Percentage of responses indicating the level of agreement regarding the topics to be discussed with the caregivers of children with myopia.

Figure 5 shows participants’ level of agreement with various topics to be discussed with the caregiver of children with myopia. Overall, the majority of participants either agreed or strongly agreed with the discussion topics listed in the figure. The long-term risks of disease associated with myopia were more likely to be explained by early career participants (OR 2.62, 95% CI 1.20–5.71, *p* = 0.016) and those with a clinical interest in managing childhood myopia (OR 3.49, 95% CI 1.15–10.64, *p* = 0.028). Similarly, management options other than spectacles were more likely to be discussed by early career participants (OR 2.12, 95% CI 1.05–4.28, *p* = 0.037) and those with a clinical interest (OR 3.12, 95% CI 1.02–9.57, *p* = 0.047). Regarding the management approach, most participants (*n* = 56) indicated that the adult caregiver was responsible for the final decision after considering the practitioner’s opinion while some participants (*n* = 29) indicated that it was a joint decision between the adult caregiver and the practitioner.

**Figure 6:**
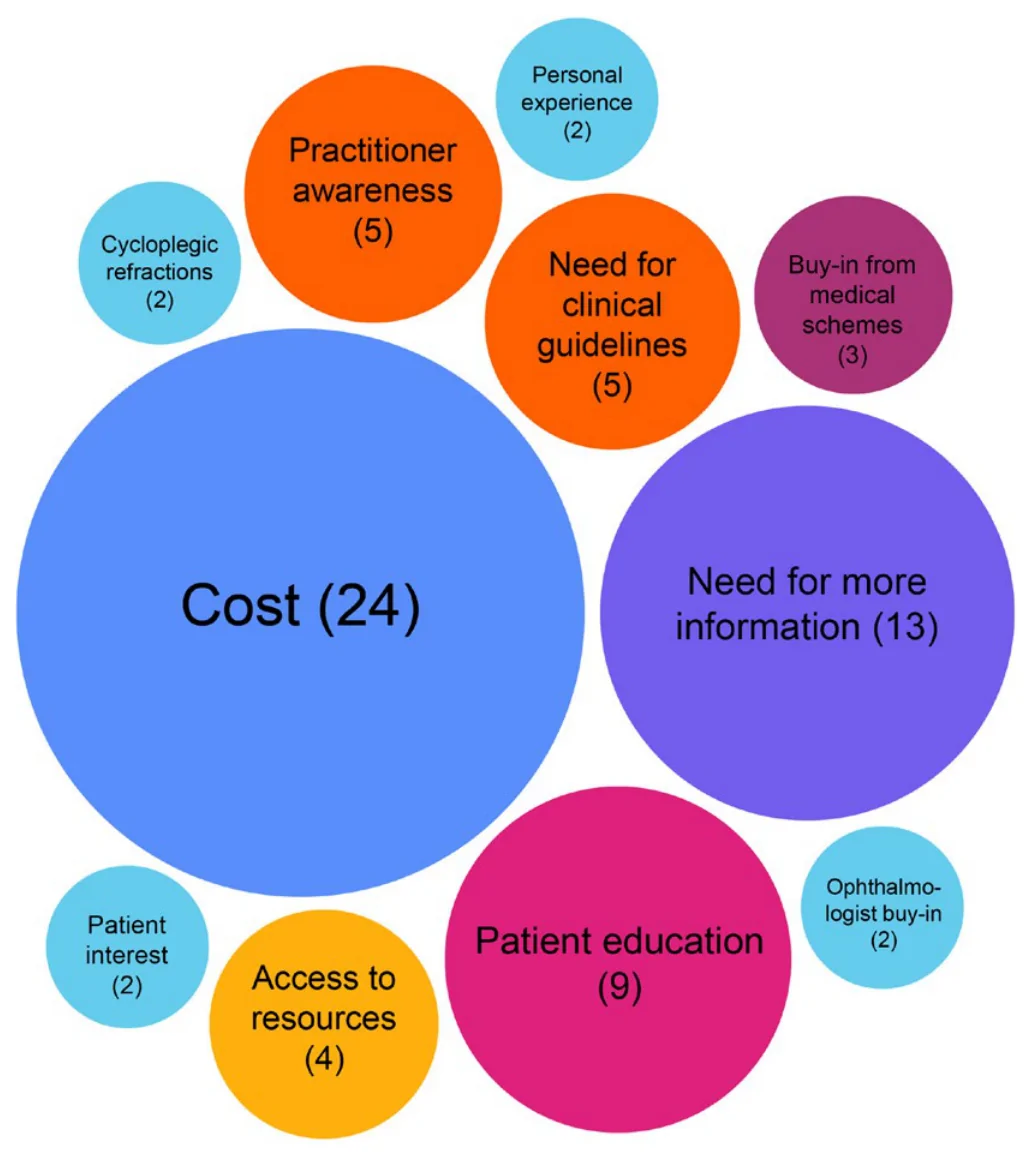
Key aspects considered to be influential in myopia management.

Figure 6 represents the key aspects participants considered influential in myopia management, with the size of the circles representing the frequency of responses for each theme. Many participants (*n* = 24) indicated that cost of myopia management was a barrier for patients. Some participants indicated that more information about myopia management (*n* = 13) is required and that there is a need for clinical guidelines (*n* = 5). One participant stated, ‘I would like there to be more seminars and colleges getting together to discuss myopia control’ (P78) while another stated ‘I think it will be helpful if we have a guideline in South Africa in how we managed myopia’ (P1). Patient education (*n* = 9) and practitioner awareness (*n* = 5) were also considered influential in myopia management. One participant stated, ‘myopia management is made difficult by lack of compliance from the guardian’ (P79) and another stated ‘…more should be invested into educating optoms (optometrists) both and (at) undergraduate and postgrad (postgraduate) level’ (P38). Less-influential factors included buy-in from medical schemes (*n* = 3) and ophthalmologists (*n* = 2). One participant stated, ‘medical aid schemes need to also allocate benefits for myopia control’ (P119).

## 4. Discussion

**Figure 7:**
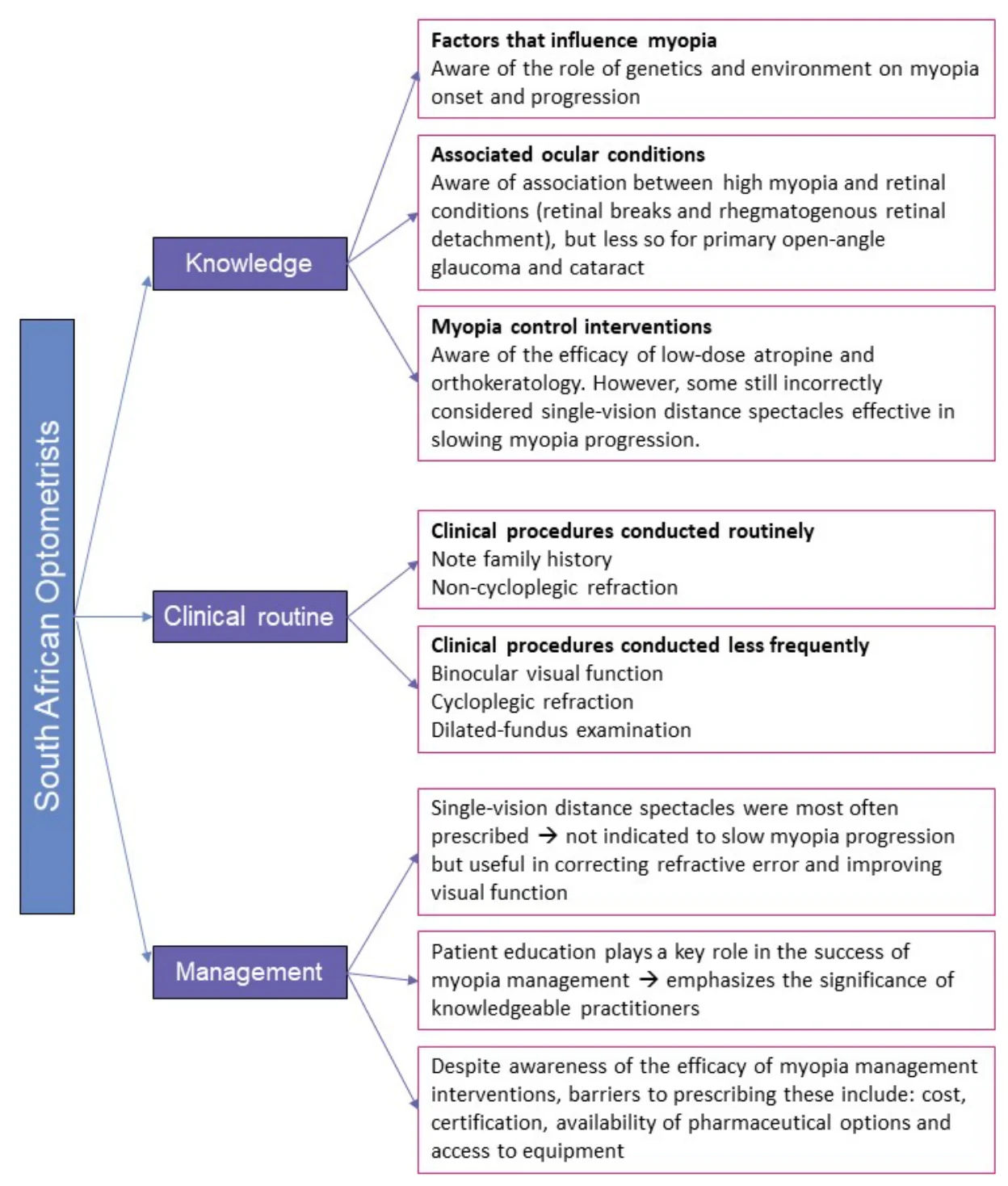
Summary of the key findings of the study.

Previous global studies that reported on knowledge and practices of eyecare practitioners towards childhood myopia management received an inadequate response rate from Africa, including South Africa, resulting in this continent being excluded from further analysis. This study aimed to fill this gap by providing baseline data on the knowledge and practices of South African optometrists regarding childhood myopia management. The rapid urbanisation of Africa coupled with the link between urbanisation and increasing myopia prevalence necessitate research into myopia management knowledge and practices of South African optometrists. However, the response rate in the present study was low, which could influence the results. Participants in this study were predominantly younger practitioners with an interest in myopia management, which could introduce potential bias. Consequently, the results of this study may overestimate the knowledge and practices of myopia management compared with the wider profession within South Africa. In addition, as the calculated sample size was not reached, the statistical power may be reduced implying that the results should be interpreted with caution. The key findings of the study are discussed below and summarised in Figure 7.

### 4.1. Participant Demographics

In this study, the majority of participants worked in independent (private) practice, which is similar to two studies [9,14] and in contrast to two studies [13,19] where the majority of participants worked in hospitals. Two universities that offer the Bachelor of Optometry degree are located in KwaZulu-Natal and Gauteng possibly accounting for the majority of participants being from these provinces. Although the expanded scope of optometry to include ocular therapeutics in South Africa was introduced in 2015, there have been challenges with completing the certification [20], which may explain why less than one quarter of participants in this study were ocular-therapeutics-certified. This is in contrast to the majority of participants who were certified for ocular diagnostics and is likely as this is currently included in the Bachelor of Optometry undergraduate degree curriculum in universities. The majority of participants who received myopia management education during their undergraduate studies were aged 20 to 39 years, which may explain the increased interest in the field of myopia management in recent years.

### 4.2. Clinical Routine and Diagnosis of Childhood Myopia

The majority of participants noted family history of myopia at a child’s initial presentation similar to other studies [9,13,14,21]. This finding indicates that South African optometrists are aware of the importance of genetic predisposition in myopia. When measuring refractive error, the majority of participants performed non-cycloplegic refraction, similar to previous studies [9,14]. In this study, almost a third of participants performed at least one cycloplegic refraction technique which is in contrast to Douglass et al. [9] where less than 20% of participants performed cycloplegic refractions. The frequency of cycloplegic refractions in these two studies is still lower than the reported frequency in other studies where the majority of participants performed cycloplegic retinoscopy at the initial presentation [19] and a cycloplegic refraction at least once every year [13]. This disparity may be related to increasing awareness more recently of the importance of a cycloplegic refraction before commencing myopia management and implies that South African optometrists are adopting this technique as standard of care in myopia practice.

A dilated-fundus examination was performed by less than 20% of participants, similar to two studies [9,14] and in contrast to one study [13], despite the majority of participants in this study being certified to conduct ocular-diagnostic techniques. Therefore, this finding could not be due to lack of diagnostic certification. Rather, possible explanations for dilated-fundus examinations not being routinely performed could be the extra chair-time required, lack of reimbursement and cost of equipment required. A minority of participants measured axial length at the initial presentation, similar to their counterparts in Australia and Spain [9,14]. This finding may be attributed to the high cost of acquiring the necessary equipment, limited clinical guidelines or poor understanding of the importance of axial length measurements. However, these results do not imply a lack of importance of ocular health assessments as the majority of participants in the present study routinely refer patients to ophthalmologists for ocular health assessments.

A notable finding in this study is that less than 65% of participants assessed binocular visual function at the initial presentation, as opposed to other studies [9,13,14,19] where the majority of participants performed at least a cover test. Research shows that the accommodative–convergence to accommodation (AC/A) ratio is greater in children prior to myopia onset than in emmetropic children [22,23]. While a higher AC/A ratio is also associated with a greater lag of accommodation, it has not been shown to influence myopia progression [22,23]. Even though there is currently no established causal link between accommodation and binocular vision with myopia development and progression, it is still necessary to assess the binocular visual status to provide patients with clear and comfortable binocular vision [22]. Furthermore, the binocular status in patients may be affected by some of the optical myopia control interventions [22]. Only a few participants measured peripheral refraction, similar to previous studies [9,13,19,21]. A study that measured relative peripheral refraction along the horizontal visual field at baseline and over two years in Chinese children found that those who developed myopia did not have more relative peripheral hyperopia at baseline compared with those who did not develop myopia [24]. Even though research shows that peripheral hyperopic defocus influences the development and progression of myopia [25], some studies showed no significant effect of baseline peripheral refraction on the onset of myopia postulating that hyperopic relative peripheral refraction may therefore be a consequence of myopia rather than a precursor to its development [26]. This implies that peripheral refraction in isolation may not be a useful indicator of the risk of developing myopia.

### 4.3. Ocular Complications Associated with High Myopia

Of the eight ocular conditions listed in the study questionnaire, only four have been found to be associated with high myopia. The majority of participants correctly identified retinal breaks and rhegmatogenous retinal detachment as being associated with high myopia, similar to other studies [9,13,19]. The current literature indicates that the risk of certain ocular diseases, particularly those affecting the posterior segment of the eye, is increased in the presence of myopia [27]. This risk increases with high degrees of myopia as a result of the axial elongation that stretches the retina, weakening it and making it susceptible to breaks and degenerative changes [28]. Furthermore, retinal disorders, like retinal breaks and rhegmatogenous retinal detachment, are commonly associated with myopia and the risk increases with high myopia [29]. Notably, while lower degrees of myopia (1.00 to 3.00 D) are associated with a four-times increased risk of developing rhegmatogenous retinal detachment, myopia greater than 3.00 D has a tenfold increased risk [27,30]. More than half the participants indicated an association between myopia and primary open-angle glaucoma, in contrast to their counterparts in Saudi Arabia and India [13,19] where less than 30% of participants selected this option. A positive association between myopia and primary open-angle glaucoma has been noted, with longer axial lengths associated with a higher prevalence of primary open-angle glaucoma [31].

In this study, less than 50% of participants indicated an association between cataract and myopia, similar to their counterparts in India [13]. A strong association has been shown to exist between myopia and posterior subcapsular cataract but not cortical cataract [31]. An association was also found between myopia and nuclear sclerotic cataract; however, this is confounded by the myopic shift that usually occurs with a nuclear-sclerotic cataract [31]. In the questionnaire used in this study, the type of cataract was not specified, which may explain the findings related to cataracts being associated with high myopia. Only a few participants were able to correctly identify all the ocular conditions associated with myopia, despite the majority of participants indicating either clinical or research interests in managing childhood myopia, similar to other studies [9,13].Considering the sight-threatening ocular conditions that arise even with low levels of myopia, it is imperative that optometrists are aware of these conditions and regularly perform dilated fundus examinations to monitor the ocular health of their patients.

### 4.4. Management of Childhood Myopia

In this study, advice on visual hygiene and time spent outdoors were considered effective in slowing childhood myopia progression, consistent with other studies [9,13,14]. It is well recognised that environmental factors play an important role in the development and progression of myopia with some of these factors including time spent outdoors, duration of near work and distance of near-work tasks [32]. Research shows a strong association between increased outdoor time and reduced myopia incidence [32]. This suggests that increased time outdoors has a protective effect, with stronger evidence for preventing the onset of myopia than for slowing its progression [32]. Zhang et al. [3] compared the incidence of myopia and lifestyle in two cohorts of children, with one pre-COVID-19 cohort and one recruited during the COVID-19 pandemic. The study found a higher incidence of myopia and a significant increase in spherical equivalent refraction and axial length at the 8-month follow-up in the COVID-19 cohort. The authors postulated that the exponential increase in screen time and decrease in time spent outdoors during the COVID-19 pandemic may have contributed to the findings [3]. This emphasises the importance of increasing time spent outdoors and good visual hygiene by limiting screen time in curbing myopia prevalence. The results of this study show that South African optometrists are aware of both the protective effect of increasing time spent outdoors and the detrimental effects of increased screen time on myopia, hence many include these options in their management of childhood myopia.

Regarding the pharmaceutical options, low-dose atropine was considered effective by approximately 75% of participants, similar to Douglass et al. [9], but in contrast to Di Pierdomenico et al. [14]. Low-dose atropine is effective in controlling myopia progression by slowing axial elongation with fewer ocular side effects [33]. While high-dose atropine has also been shown to slow axial elongation in myopia, this option is not viable because of the greater severity of ocular side effects [33]. In addition, the rebound effect that occurs after cessation of high-dose atropine use results in acceleration of myopia progression that negates any prior treatment effect [34]. The results of this study showed that even though most South African optometrists are aware of the efficacy of low-dose atropine, only a few optometrists prescribe it. Some of the possible reasons for this may be limited availability and the need to compound atropine eye drops to the required dosage as well as a lack of the necessary certification to prescribe atropine. This highlights the need to address the challenges and streamline the process by which optometrists may obtain therapeutics certifications. Improving the therapeutics-certification process would be advantageous to the general public as it would allow for improved access to essential eyecare services as well as reduce barriers, such as costs. In addition, despite being used for myopia control elsewhere, participants may be wary of the legal implications of prescribing atropine eye drops off-label as currently there are no approved pharmacological options by the Food and Drug Administration (FDA) and the South African Health Products Regulatory Authority (SAHPRA) for myopia control [33,35,36].

Orthokeratology was considered effective by most participants in this study similar to one study [9] but in contrast to four other studies [11,13,14,21]. In a large-scale global study, practitioners perceived orthokeratology as the most effective myopia control intervention [8]. Similarly, in the most recent global survey, orthokeratology was ranked second to combination therapy as the most effective myopia control intervention [15]. Orthokeratology has been shown to significantly reduce axial elongation, with efficacy ranging from 32% to 63%, relative to single-vision correction [37]. The results of this study showed that although participants are aware of the efficacy of orthokeratology in myopia control, only a few regularly prescribed this option. Given that orthokeratology is a specialised process, optometrists would require additional training likely explaining the low number of participants who regularly prescribe it. The lack of necessary training for orthokeratology and certification for low-dose atropine would also have implications for prescribing combination therapy. Combination therapy, which includes more than one type of myopia control intervention, has been found to be more effective in slowing myopia progression than any intervention in isolation [38]. The superior efficacy of this modality is especially useful in younger children and those with a faster rate of progression [38]. Endorsing orthokeratology training among optometrists and improving access to pharmaceutical interventions would improve the prescription of combination therapy using these modalities. That almost 40% of participants considered single-vision distance spectacles effective in slowing myopia progression is concerning considering that they have no effect in slowing myopia progression and, based on results from animal studies, may exacerbate axial elongation [25]. One possible explanation for this may be inadequate differentiation between the terms ‘myopia correction’ and ‘myopia control’ among optometrists with these terms sometimes being used interchangeably [6].

Single-vision distance spectacles were the most commonly prescribed modality, consistent with other studies [9,11,12,13,14,19,21]. In the most recent global survey by Wolffsohn et al. [15], single-vision spectacles were most commonly prescribed in all regions except Australasia; however, there has been a decrease since the previous global survey [8]. This trend may be explained by the high cost and availability of myopia control interventions, as well as lack of awareness regarding myopia control. One study explored the rate of myopia progression between uncorrected myopic children and those with single-vision spectacles [39]. Contrary to previous research, this study found no difference in the rate of myopia progression between both groups, indicating that single-vision spectacles neither increase nor decrease myopia progression [39]. The authors stated that single-vision spectacles may be prescribed to children if the visual reduction caused by myopia affected performance of daily tasks. However, the results of this study should be interpreted with caution as the study included only Chinese children with low myopia, with clinical examination including only non-cycloplegic autorefraction and no axial-length measurements. Nonetheless, the results of that study may be useful in instances where single-vision correction of myopia is considered especially when there are barriers related to affordability of appropriate myopia control interventions. In this way, correcting the refractive error would improve functional vision positively impacting educational and avocational activities thereby enhancing quality of life [40].

### 4.5. Factors That Influence the Management of Childhood Myopia

In the current study, factors that influenced the management of childhood myopia included rate of myopia progression, current refractive error, patient’s age and amount of time spent undertaking near work, similar to a previous study [9]. The rate of myopia progression is influenced by the age of the child and current refractive error, with younger age and higher levels of myopia associated with faster progression [41]. Furthermore, earlier onset of myopia is associated with faster rates of progression and greater severity in adulthood [5,41]. The literature supports that appropriate education of children with myopia and their parents/caregivers is a key factor in the success of myopia management [42]. Patient education that is consistent, comprehensive, personalised and includes the long-term risks of myopia is necessary to increase awareness of this public-health problem [43]. In this study, most participants indicated that the parent/caregiver made the final decision regarding management of the myopic child after considering the practitioner’s opinion. This highlights the significant role that eye-care practitioners play in patient education and further emphasises the need for them to be knowledgeable about the latest developments in myopia-control interventions.

In this study, the need to purchase additional clinical equipment and minimal financial incentive were two major factors that were considered to limit the management of childhood myopia, similar to two other studies [10,21]. In addition, participants cited cost to the patient as a major barrier, which is consistent with previous studies [10,11,12,15,44]. The financial burden on the patient is especially relevant in a developing country like South Africa where basic living costs, such as housing, utilities, transport and food, may take precedence over health care. Lack of regulatory approval of treatments and concern over medico-legal aspects of prescribing interventions other than spectacles were also considered to be limiting factors. Although research shows various myopia control interventions to be successful in slowing myopia progression, some of these, such as low-dose atropine and repeated low-level red-light therapy, are off-label [35], which may be a concern for some practitioners. This concern may be alleviated by practitioners providing sufficient information about the latest evidence-based interventions to parents/caregivers in order for them to make an informed decision and is especially important when recommending an intervention that is off-label [35]. Furthermore, considering the risks and benefits of myopia control for a child with myopia is useful when making a recommendation about any specific intervention [35].

Other aspects considered influential by participants are the need for clinical guidelines and improved practitioner awareness. This is highlighted by the lack of consensus regarding the appropriate age and duration of myopia stability at which to cease active treatment for a patient. Other studies also reported a lack of clinical guidelines and inadequate information as barriers to myopia management [8,9,10,11,12,44]. In the most recent global survey, inadequate information was of greater concern among practitioners from Africa compared to elsewhere [15]. Much research has been done on myopia management, including studies related to myopia control interventions. However, the vast amount of research available may be overwhelming and time-consuming for practitioners to review making it difficult to keep abreast with the latest developments, which may explain this result. This is similar to a study that conducted focus-group discussions with eye-care practitioners from the United Kingdom where participants reported difficulty keeping up with the latest research [44]. In this study, the majority of participants reported relying on continuing education events and conferences for information related to myopia management. This is noteworthy as continuing education events and conferences often summarise and deliver information on the latest developments in a more manageable format for practitioners. The results of this study indicate the need for a localised, contextually relevant implementation protocol that is comprehensive and user-friendly to minimise practitioner uncertainty related to management of childhood myopia.

### 4.6. Strengths and Limitations

This study adds to the body of knowledge regarding the practice of childhood myopia management and provides information from a South African perspective, which is currently limited as results from only one regional study are currently available. Although this study used a questionnaire that has been validated in previous studies, tests for reliability and validity were not conducted in the present study. One of the limitations of this study could be the low response rate, which has also been reported in previous studies. This may be due to survey fatigue as a result of the increase in myopia-related research in recent years. Another possible limitation could be volunteer bias as many of the participants in this study were younger practitioners with either a clinical or research interest in myopia management, which could have influenced their decision to participate in the study. Therefore, the results of this study possibly overestimate the knowledge and uptake of myopia management and may not be fully representative of South African optometrists. Despite this, the results of this study are useful as they provide baseline data that may be used in future studies.

## 5. Conclusions

This study provides baseline information on the knowledge and practices of the study sample and may not necessarily reflect the knowledge and practices of the wider population of South African optometrists. Overall, there is inconsistency in the clinical procedures routinely performed on a myopic child, particularly cycloplegic refraction and dilated-fundus examinations. In addition, there is limited awareness of the ocular conditions associated with high myopia, underscoring the need for greater knowledge especially considering the sight-threatening nature of these conditions. Despite awareness of the efficacy of myopia control interventions, single vision spectacles were most commonly prescribed to children with myopia. Furthermore, the practice of evidence-based myopia management is greatly influenced by structural barriers, such as equipment availability and cost. The results of this study may be used to guide future optometry policy development and revision, as well as, regulatory council decisions related to the examination and management of childhood myopia. These may include streamlining the process by which optometrists achieve therapeutics certification as well as advocating and negotiating for the affordability of myopia control interventions. These results may also be used to guide the content of professional development programs to enhance the knowledge of practicing optometrists.

## Figures and Tables

**Table 1:** Demographic information.

Characteristic	Frequency (%)
Gender	
Female	92 (74.2)
Male	31 (25.0)
Prefer not to say	1 (0.8)
Age range	
20–29 years	53 (42.7)
30–39 years	39 (31.5)
40–49 years	17 (13.7)
50–59 years	13 (10.5)
60–69 years	2 (1.6)
Optometric practice experience	
0–5 years	44 (35.5)
6–10 years	29 (23.4)
11–15 years	12 (9.7)
16–20 years	14 (11.3)
>20 years	25 (20.2)
Primary place of practice	
Academic institution	7 (5.6)
Corporate practice	33 (26.6)
Hospital	7 (5.6)
Independent (private) practice	73 (58.9)
Locum	1 (0.8)
Ophthalmologists’ rooms	1 (0.8)
Public health clinic	2 (1.6)
Location of primary place of practice	
Rural	25 (20.2)
Urban	99 (79.8)
Location of primary place of practice (province)	
Eastern Cape	15 (12.1)
Free State	6 (4.8)
Gauteng	38 (30.6)
KwaZulu-Natal	48 (38.7)
Limpopo	3 (2.4)
North West	2 (1.6)
Northern Cape	1 (0.8)
Western Cape	11 (8.9)
Ocular diagnostic techniques certified	
Yes	109 (87.9)
No	15 (12.1)
Ocular therapeutics certified	
Yes	24 (19.4)
No	100 (80.6)
Clinical interest in managing childhood myopia	
Yes	113 (91.1)
No	11 (8.9)
Research interest in managing childhood myopia	
Yes	87 (70.2)
No	37 (29.8)
Number of myopic patients under 18 years of age provided care per week	
0–5	74 (59.7)
6–10	33 (26.6)
11–15	13 (10.5)
16–20	2 (1.6)
21–25	0
26–30	0
>30	2 (1.6)

**Table 2:** Clinical procedures performed routinely on a child with myopia at initial presentation.

Clinical Procedure	Frequency (%)
Note family history of myopia	118 (95.2)
Non-cycloplegic subjective refraction	100 (80.6)
Intraocular pressure	94 (75.8)
Non-cycloplegic retinoscopy	87 (70.2)
Retinal fundus photography—posterior pole	85 (68.5)
Non-cycloplegic autorefraction	84 (67.7)
Measurement of distance phoria	80 (64.5)
Measurement of near phoria	79 (63.7)
Dynamic retinoscopy (e.g., MEM retinoscopy)	56 (45.2)
AC/A ratio determination	53 (42.7)
Cycloplegic retinoscopy	45 (36.3)
Stereopsis	45 (36.3)
Retinal fundus photography—peripheral	39 (31.5)
Cycloplegic subjective refraction	32 (25.8)
Measurement of pupil size	32 (25.8)
Corneal topography	31 (25.0)
Cycloplegic autorefraction	28 (22.6)
Dilated retinal fundus examination	22 (17.7)
Axial length measurement	9 (7.3)
Peripheral refraction	4 (3.2)
Other	6 (4.8)

AC/A, accommodative convergence/accommodation; MEM, monocular estimation method.

**Table 3:** Frequency of responses regarding ocular conditions associated with high myopia.

Ocular Condition	Frequency (%)
* Retinal breaks	106 (85.5)
* Rhegmatogenous retinal detachment	93 (75)
* Primary open-angle glaucoma	70 (56.5)
* Cataract	60 (48.4)
Exudative retinal detachment	28 (22.6)
Age-related macular degeneration	22 (17.7)
Primary angle closure glaucoma	22 (17.7)
Diabetic retinopathy	2 (1.6)

* indicates an established association with high myopia.

**Table 4:** Frequency of responses regarding the efficacy of myopia control interventions.

Myopia Control Option	Frequency (%)
Advice to increase time spent outdoors	112 (90.3)
Low-dose (0.01–0.5%) atropine eye drops	94 (75.8)
Visual hygiene	91 (73.4)
Orthokeratology	83 (66.9)
Peripheral-defocus soft-contact lenses	82 (66.1)
Progressive-addition spectacle lenses	70 (56.5)
Bifocal spectacle lenses	48 (38.7)
Single-vision distance spectacles (full correction)	48 (38.7)
Cyclopentolate 1% eye drops	23 (18.5)
Single-vision distance spectacles (under-correction)	13 (10.5)
Bifocal spectacle lenses with prism	12 (9.7)
High-dose (>0.5%) atropine eye drops	11 (8.9)
Other	20 (16.1)

## Data Availability

All relevant data have been provided in the manuscript. The dataset used in the current study is available from the corresponding author, S.N., upon reasonable request due to privacy reasons.

## Supplementary Materials

The following supporting information can be downloaded at: https://www.mdpi.com/article/10.3390/vision10040063/s1, Figure S1: Percentage of responses regarding the relative importance of various factors that limit the management of childhood myopia; Table S1: Frequency (%) of responses for clinical scenario regarding age and duration of myopia stability to cease treatment of myopia; Figure S2: Percentage of responses regarding the relative importance of various resources in guiding practitioners’ approach to managing childhood myopia; Figure S3: Percentage of responses regarding the relative usefulness of resources in supporting clinical management of childhood myopia; Table S2: Regression analysis results for myopia management based on practitioner experience, certification and interest.

## Abbreviations

The following abbreviations are used in this manuscript:

AC/A	Accommodative convergence to accommodation
CI	Confidence interval
CL	Contact lenses
FDA	Food and drug administration
HPCSA	Health Professions Council of South Africa
MEM	Monocular estimation method
OR	Odds ratio
SAHPRA	South African Health Products Regulatory Authority
SPSS	Statistical Package for Social Sciences
SVD	Single vision distance
WCO	World Council of Optometry
