# Supplementary material for: Knowledge and Practices of South African Optometrists Regarding Childhood Myopia Management
Source: Vision (Basel). 2026 Sep 1;10(4):63. doi: 10.3390/vision10040063 (PMC13600011; doi:10.3390/vision10040063)
Supplement: Supplementary file 1 [file vision-10-00063-s001.zip › vision-4471493-supplementary.pdf]

Supplementary Materials: Knowledge and practices of South African optometrists regarding childhood myopia management

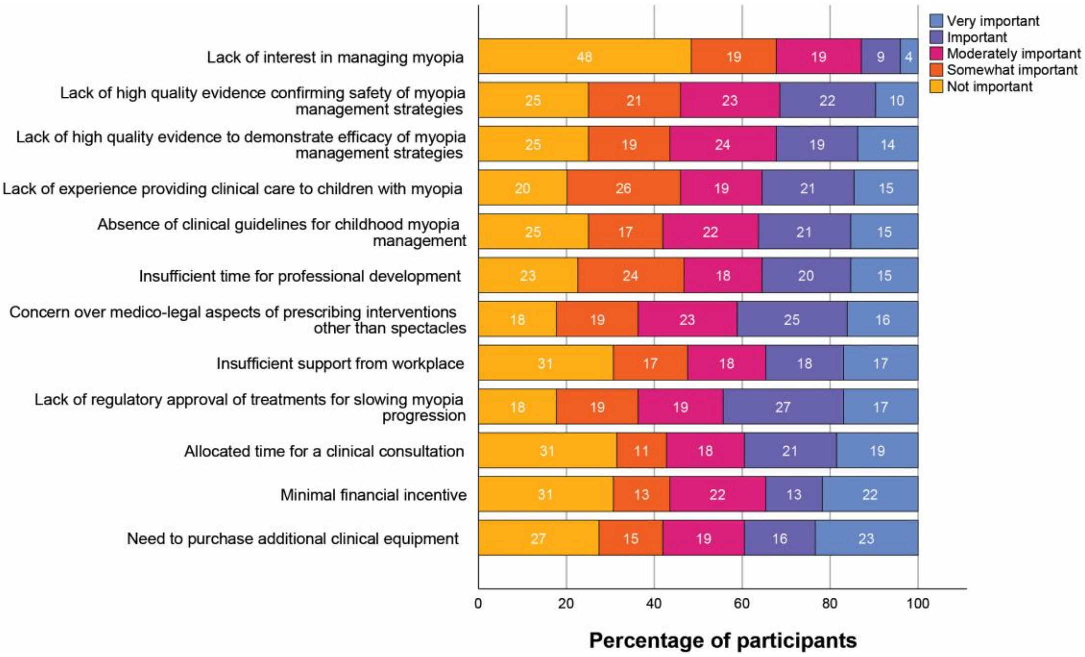

Supplementary Figure S1: Percentage of responses regarding the relative importance of various factors that limit the management of childhood myopia

Supplementary Table S1: Frequency (%) of responses for clinical scenario regarding age and duration of myopia stability to cease treatment of myopia

	Duration of myopia stability					
	**6 months**	**12 months**	**18 months**	**24 months**	**Progression rate not important**	**Total**
**16 years**	2 (1.6)	9 (7.3)	0	8 (6.5)	1 (0.8)	20 (16.1)
**18 years**	1 (0.8)	10 (8.1)	5 (4.0)	12 (9.7)	2 (1.6)	30 (24.2)
**21 years**	2 (1.6)	8 (6.5)	4 (3.2)	22 (18.0)	1 (0.8)	37 (29.8)
**25 years**	0	4 (3.2)	3 (2.4)	7 (5.6)	0	14 (11.3)
**Any age**	1 (0.8)	6 (4.8)	4 (3.2)	8 (6.5)	4 (3.2)	23 (18.5)
**Total**	6 (4.8)	37 (29.8)	16 (12.9)	57 (46.0)	8 (6.5)	124 (100)

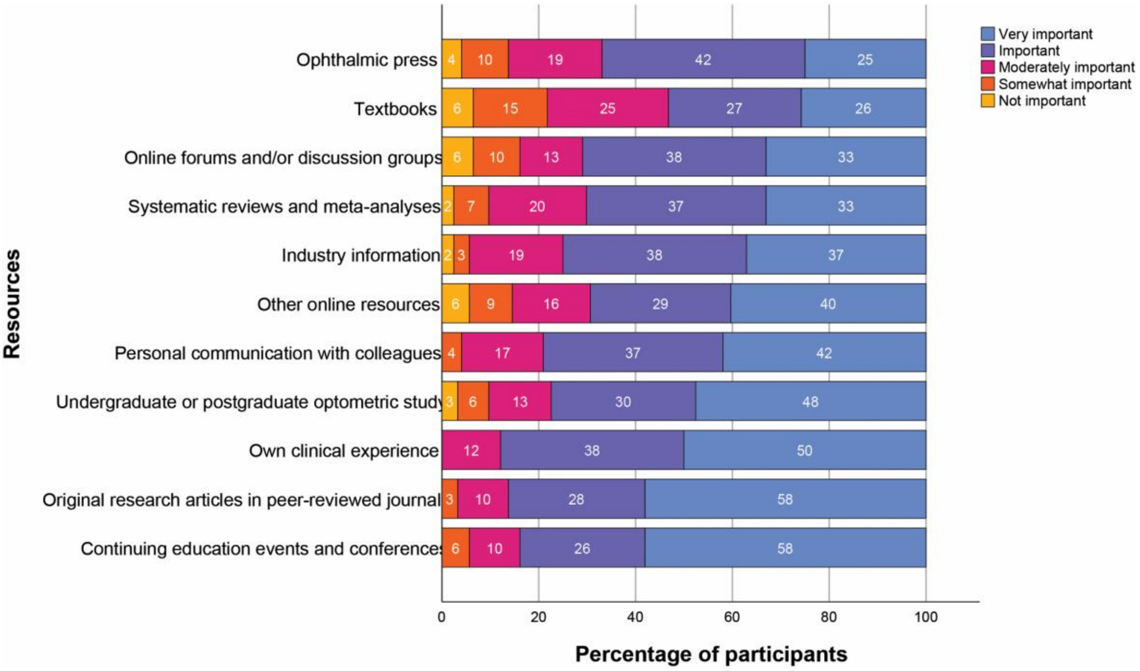

Supplementary Figure S2: Percentage of responses regarding the relative importance of various resources in guiding practitioners' approach to managing childhood myopia

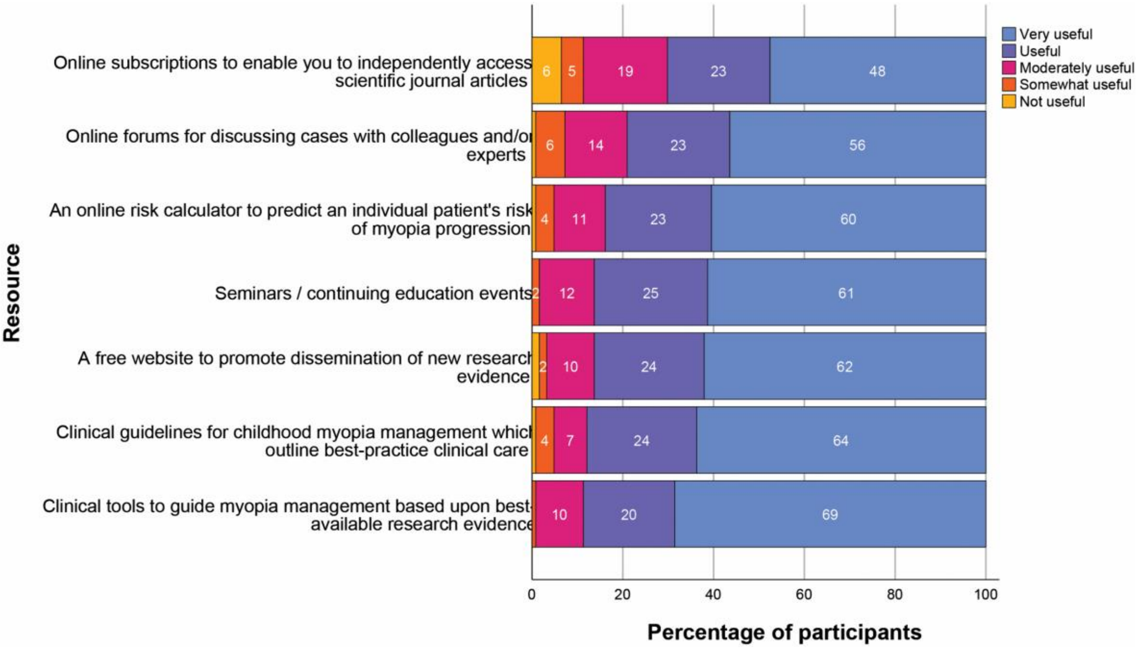

Supplementary Figure S3: Percentage of responses regarding the relative usefulness of resources in supporting clinical management of childhood myopia

Supplementary Table S2: Regression analysis results for myopia management based on practitioner experience, certification and interest

Categories	Experienced practitioners			Diagnostics certified			Therapeutics certified			Clinical interest			Research interest		
	OR	95% CI	p-value	OR	95% CI	p-value	OR	95% CI	p-value	OR	95% CI	p-value	OR	95% CI	p-value
**Clinical procedures performed at a child's first presentation**															
Non-cycloplegic retinoscopy	2.22	1.01-4.90	0.048	1.68	0.55-5.11	0.363	0.82	0.32-2.12	0.677	0.21	0.03-1.74	0.149	0.99	0.43-2.30	0.986
Intraocular pressure	0.59	0.24-1.46	0.249	3.27	1.07-9.97	0.037	1.27	0.43-3.75	0.669	0.68	0.14-3.31	0.628	1.24	0.51-2.99	0.631
**Clinical procedures routinely conducted**															
Manifest subjective refraction	0.64	0.33-1.24	0.184	1.26	0.45-3.54	0.667	2.03	0.90-4.59	0.090	5.76	1.96-17.29	0.002	1.99	1.00-3.96	0.049
**Ocular complications associated with high myopia**															
Primary open angle glaucoma	0.85	0.40-1.78	0.660	6.38	1.70-23.95	0.006	0.59	0.24-1.44	0.246	2.46	0.68-8.88	0.170	1.83	0.84-3.99	0.126
Retinal breaks	0.90	0.31-2.58	0.837	3.69	1.09-12.50	0.036	0.81	0.24-2.74	0.739	1.35	0.27-6.81	0.719	0.89	0.29-2.70	0.836
**Perceived efficacy of myopia control interventions**															
Low dose atropine	0.72	0.30-1.75	0.472	3.27	1.07-9.97	0.037	0.95	0.34-2.66	0.918	1.19	0.30-4.82	0.803	0.82	0.33-2.05	0.663
Orthokeratology	0.78	0.35-1.72	0.537	1.93	0.65-5.75	0.238	0.63	0.25-1.57	0.321	6.47	1.61-25.9	0.008	1.89	0.85-4.20	0.119
Peripheral defocus soft CL	1.19	0.55-2.57	0.664	3.46	1.14-10.49	0.029	0.82	0.33-2.07	0.676	6.20	1.55-24.79	0.010	1.52	0.68-3.37	0.308
Progressive addition spectacle lenses	2.31	1.09-4.90	0.029	1.57	0.53-4.62	0.418	2.15	0.82-5.65	0.119	0.72	0.20-2.60	0.616	1.34	0.62-2.91	0.456
SVD spectacles (full correction)	0.42	0.20-0.89	0.023	0.69	0.23-2.04	0.501	0.26	0.08-0.80	0.019	0.74	0.21-2.56	0.631	0.90	0.41-1.97	0.785
**Factors influencing management**															
Parents' refractive status	0.29	0.14-0.60	0.001	1.90	0.75-4.85	0.177	0.74	0.31-1.74	0.486	4.68	1.53-14.28	0.007	1.90	0.92-3.01	0.081
Time on near work	0.53	0.25-1.13	0.098	1.15	0.42-3.20	0.785	0.43	0.19-0.98	0.045	3.05	1.07-8.66	0.037	2.08	1.00-4.29	0.049
Patient ethnicity	0.79	0.41-1.53	0.485	1.16	0.44-3.05	0.760	0.36	0.16-0.81	0.013	4.20	1.23-14.33	0.022	1.66	0.83-3.33	0.151
**Factors that limit management**															
Lack of interest	1.30	0.64-2.63	0.473	0.84	0.29-2.46	0.748	1.44	0.64-3.26	0.377	0.16	0.05-0.48	0.001	0.38	0.19-0.78	0.008
Need to purchase equipment	0.33	0.17-0.64	0.001	0.44	0.16-1.25	0.123	0.61	0.29-1.29	0.194	0.48	0.15-1.53	0.215	1.24	0.62-2.47	0.541
Minimal financial incentive	0.36	0.18-0.71	0.003	0.72	0.27-1.91	0.511	0.78	0.36-1.69	0.529	0.73	0.26-2.03	0.548	1.59	0.80-3.14	0.185
**Management options prescribed for childhood myopia**															
Peripheral aberration control	1.01	0.52-1.96	0.968	2.67	0.98-7.26	0.054	2.65	1.17-6.01	0.019	6.70	1.75-25.6	0.005	1.35	0.68-2.70	0.392
Low dose atropine	0.98	0.45-2.13	0.962	2.09	0.56-7.75	0.270	2.61	1.10-6.19	0.030	1.49	0.39-5.72	0.562	0.87	0.39-1.93	0.736

Increase outdoor time	0.61	0.27-1.37	0.233	1.36	0.47-3.93	0.569	0.81	0.31-2.07	0.653	3.00	0.94-9.54	0.064	3.45	1.57-7.56	0.002
**Sources of information that influence perceptions of myopia management intervention efficacy**															
Systematic review & meta-analyses	0.52	0.21-1.26	0.147	3.95	0.49-31.6	0.195	0.75	0.23-2.44	0.635	2.70	0.33-22.1	0.356	3.84	1.07-13.7	0.039
**Engagement with caregivers**															
Long-term risks	0.38	0.18-0.83	0.016	1.67	0.64-4.37	0.298	1.45	0.58-3.65	0.425	3.49	1.15-10.6	0.028	1.53	0.73-3.21	0.263
Options other than spectacles	0.47	0.23-0.96	0.037	1.54	0.59-4.05	0.380	1.87	0.77-4.54	0.166	3.12	1.02-9.57	0.047	1.48	0.71-3.08	0.298

OR: odds ratio; CI: confidence interval; CL: contact lenses; SVD: single vision distance. The cells shaded in blue represent the variables with acceptable goodness-of-fit parameters.
